# Surveillance in Barrett Esophagus

**Published:** 2014

**Authors:** C Gindea, R Birla, P Hoara, A Caragui, S Constantinoiu

**Affiliations:** * “Carol Davila” University of Medicine and Pharmacy, Bucharest; “Sf. Maria” Clinical Hospital, General and Esophageal Surgery Department, Bucharest, Romania

## Abstract

The only known precursor of the esophageal adenocarcinoma (EAC) is represented by the Barrett’s esophagus (BE). EAC incidence has increased sharply in the last 4 decades.

The annual conversion rate of BE to cancer is small but significant; therefore the identification of patients at a higher risk of cancer represents a dilemma.

The endoscopic surveillance of BE aims to detect dysplasia and in particular high-grade dysplasia and intramucosal cancers that can be endoscopically treated before progressing to invasive cancer with lymph node metastases.

Using standard white light endoscopy (WLE), these high-risk lesions are often subtle and hard to detect.

In addition to high-definition standard endoscopy, chromoendoscopy (CE), virtual chromoendoscopy (e.g. narrow band imaging), and confocal laser endomicroscopy might increase the diagnostic efficiency for the detection of dysplastic lesions and can also increase the diagnostic efficiency for the detection of BE dysplasia or cancer. This ability to detect subtle mucosal abnormalities that harbor high-grade dysplasia (HGD) or intramucosal carcinoma might enable endoscopists skilled in the assessment of BE to perform targeted rather than random biopsies.

The standard protocol will remain the careful examination by using conventional high-resolution endoscopes, combined with a longer inspection time, which is associated with an increased detection of dysplasia until these modalities have been demonstrated to enhance efficiency or be cost effective.

Many of the limitations of the current clinical standard may be overcome in the future by the use of multi-modal imaging combined with molecular information.

## Introduction

Symptoms caused by gastro-esophageal reflux disease (GERD) or by esophageal cancer lead to the endoscopic evaluation of the patients and so Barrett’s esophagus (BE) is usually discovered. Studies show that in the general population, more than 90% of cases of Barrett’s esophagus are not recognized and many patients with the condition have few or no symptoms of GERD [**1**].

Because of the increased risk of malignant transformation to adenocarcinoma, BE is an indication for endoscopic surveillance. The annual risk of malignant transformation from BE to adenocarcinoma is of 0.12–0.5% [**2**].

The advantages presented by the endoscopic surveillance are that cancers occurring in patients under surveillance for BE are detected at an earlier stage, and are consequently associated with a better survival and a chance of being treated endoscopically, without the risks posed by major surgery [**3**].

**Endoscopic surveillance**

The aim of endoscopic surveillance recommended for BE patients is to diagnose either dysplasia or cancer at early stages, both of which are curable with minimally invasive endoscopic techniques. 

Based on the known clinical risk factors for BE and esogastric adenocarcinoma (EAC), an accurate algorithm to confidently target the population at risk is currently difficult to formulate. 

Surveillance in BE is also a controversial issue because even if nowadays it is generally accepted that patients with BE should be monitored over time, definitive evidence that systematic endoscopic surveillance improves survival is still lacking.

A recent large case–control retrospective study involving 8,272 individuals with BE found that endoscopic surveillance was not associated with a significant reduced risk of death from EAC [**4**].

However, the lead-time bias limits these studies. By contrast, a more recent case-control study from Corley and collaborators [**5**] has suggested that previous endoscopic surveillance has no significant impact on mortality from EAC.

Several retrospective studies showed that EAC and junctional adenocarcinomas diagnosed within a previous background of known BE have an earlier stage and an improved survival rate when compared to cancers presenting de novo [**6**,**7**].

However, the authors discovered an unusually high prevalence of advanced stage cancers in patients undergoing surveillance, suggesting that in this cohort of patients, endoscopic surveillance did not efficiently achieve the expected goal of detecting early disease.

Also in this study, there was a higher proportion of dysplasia in previous biopsies of cases that died of EAC, compared to controls that did not die of this disease. 

Even so, outside of specialized centers, there may be methodological problems with the surveillance protocols in routine practice.

All the gastroenterology societies generally accept and recommend routine surveillance; the American Gastroenterology Association (AGA) working group commented on the fact that it remains unclear whether endoscopic surveillance is beneficial, so it was not possible to make meaningful recommendations regarding the optimal intervals between endoscopic procedures [**8**]. 

Because of the well-established association between BE and EAC and the very poor outcomes of this cancer, while we wait for convincing evidence that endoscopic surveillance is beneficial, it seems necessary to survey BE patients over time. 

A multicenter U.K. based random control trial (BOSS trial) is currently being undertaken to address the long-term clinical impact of endoscopic surveillance [**9**].

In this study, patients who present BE without dysplasia are being randomized into two groups, surveillance versus no surveillance. This is hoped to provide scientific evidence that will support the practice of endoscopic surveillance.

The current gold standard represented by endoscopy with biopsies, which is both invasive and expensive, represents one of the main implications of widespread surveillance.

In order to improve the cost-effectiveness of surveillance, research is currently focused on two directions. One is represented by the development of biomarkers in order to risk stratify patients into low and high-risk individuals. The rationale is to provide a more objective assessment of the individual cancer risk to overcome the shortfalls of a pathological assessment of dysplasia.

Dysplasia, as a marker of increased risk, is affected by sampling error and high interobserver variability because of the large variation that appears in clinical practice for endoscopic surveillance [**10**]. 

In order to better exhibit the metaplastic tissue, jumbo biopsy forceps and modern techniques of endoscopy (like chromoendoscopy, autoflourescence or endomicroscopy) are used. It was demonstrated that the method sensitivity is altered when using the methylene-blue for the detection of dysplasia because of the oxidative alterations of the columnar epithelium DNA [**11**].

This would allow stretching out intervals for surveillance in low risk patients with the potential to discharge them and on the other hand anticipate the ablation treatment in high-risk patients.

**Intense Surveillance**

The intense surveillance involves endoscopic examinations of high-grade dysplasia (HGD) at every 3–6 months, with holding invasive treatments like esophagectomy until biopsy specimens reveal adenocarcinoma. Few studies support this approach, as various series have reported multiple patients with incurable disease (metastases) when the cancer was first detected on surveillance endoscopy [**12**,**13**].

AGA recommends endoscopic eradication therapy rather than surveillance as a standard treatment of patients with HGD (AGA Medical Position Panel 2011). 

**Negative Surveillance**

Frequently, negative surveillance occurs in short-segment non-dysplastic Barrett esophagus (NDBE). An initial negative surveillance endoscopy may occur either due to a sampling error or because of a lack of detectable intestinal metaplasia (IM) on the surveillance exam. When a <1 cm segment of NDBE is diagnosed, a significant proportion of patients may go on to have continuously undetected IM on consecutive surveillance endoscopic exams without intervention [**14**].

The second goal of researchers is to determine the less invasive and the most cost-effective technologies suitable for surveillance. Devices suited for surveillance would need some form of tissue collection either for pathological analysis or for biomarker assessment different from the screening devices.

Age, likelihood of survival over the next 5 years, the patient’s understanding of the process and its limitations for the detection of cancer and also the willingness of the patient to adhere to the recommendations should be considered when starting a surveillance programme, is the recommendation of the American College of Gastroenterology (ACG) [**15**].

Regular surveillance involves cost and use of resources, and such spending, while meeting healthcare system obligations, must be economically sustainable [**16**].

In addition to the high cost of endoscopy and the small risk of endoscopic complications, there are adverse consequences of endoscopic screening and surveillance. The identification of innocuous neoplastic lesions by the use of these procedures might lead to the use of invasive therapies with serious or even fatal complications. Studies have shown that a diagnosis of Barrett’s esophagus causes psychological stress as well as a negative effect on the quality of life and results in higher premiums for health and life insurance [**17**].

Currently, the medical societies state that in the absence of definitive data, it is better to make an error by performing unnecessary screening and surveillance than passing by the opportunity to identify curable esophageal neoplasms. If future recommendations will be influenced by the new data, it is still unclear. Screening and surveillance for Barrett’s esophagus are still recommended by medical societies even if they still present many limitations and dubious benefits. Recommendations for surveillance of established Barrett’s esophagus are stronger and more explicit than the recommendations for the initial screening [**18**].

**Risk stratification in BE patients**

Risk factors such as age, sex, obesity, body mass index (BMI), race, smoking habits, length of the BE segment [**18**] (more or 6–8 cm) or endoscopic signs such as ulcers or nodularity, and perhaps hiatal hernia, have been variously associated with a higher risk of progression to adenocarcinoma but, with the exception of visible lesions at endoscopy, they are widely nonspecific and common for many BE patients [**19**].

**Endoscopic markers**

Specific biological markers to help us identify BE patients at risk are not yet clinically proven, and until these become available to clinical practice, endoscopic, and pathological features could be used in order to stratify BE patients [**20**].

The extent of metaplasia significantly influences the risk for adenocarcinoma. Spechler et al. concluded that the “intestinal-type metaplasia only” definition should be discarded, in favor of the following: (Barrett's esophagus is) . . . “the condition in which any extent of metaplastic columnar epithelium that predisposes to cancer development replaces the stratified squamous epithelium that normally lines the esophagus” [**21**].

The most important identified risk factor for the esophageal adenocarcinoma is represented by the long-segment Barrett’s esophagus with the intestinal type metaplasia [**22**].

The Seattle protocol represents the current surveillance practice standard and requires the collection of random 4-quadrant biopsy specimens over every 1 to 2 cm of BE in order to detect dysplasia with the assistance of white light endoscopy (WLE), in addition to performing targeted biopsies of recognizable lesions. Despite its limitations, such as the extra time and cost to acquire and interpret the many biopsies, which consequently lead to poor adherence by physicians, it has been adopted as the standard of care [**23**,**24**].

In accordance with the Seattle protocol, multiple random biopsies should be collected because histological confirmation of IM is an essential requisite for the diagnosis of BE, as well as a tool for the stratification of patients with NDBE, low-grade dysplasia (LGD), or HGD so as to ensure further surveillance and treatment [**25**].

Visible lesions that are considered suspicious for dysplasia should be targeted separately for biopsy and should be described precisely with regard to their location, size, and macroscopic aspect. While nodular or depressed ulcerated lesions are easy to recognize, the flat and occult lesions represent an endoscopic challenge [**26**].

**Pathologic markers - Dysplasia**

Nowadays, in routine clinical practice, a patient's risk for EAC development is assessed only crudely by determining if dysplasia is absent or present and if it is present, whether it is of low or high grade [**27**].

Updated guidelines of the American College of Gastroenterology recommend that if no evidence of dysplasia is found within the Barrett’s epithelium on 2 consecutive endoscopies with biopsy, surveillance endoscopy intervals must be lengthened to a 3-year surveillance interval [**28**].

Many other professional societies adapted these recommendations.

The phenotypic sequence of the progress from BE to EA is from no dysplasia to low-grade dysplasia (LGD), then high-grade dysplasia (HGD) and finally to adenocarcinoma.

LGD states for mild diffuse cytological abnormalities such as nuclear hyperchromasia and nuclear membrane irregularities, normal nuclear polarity, mildly abnormal architecture with glandular crowding, but well identifiable basal membrane and distorted surface maturation, with a surface similar to the lower gland, while HGD presents marked cytological alterations, nuclear hyperchromasia, irregular nucleoli with loss of nuclear polarity, marked architectural alterations with crowding of cytologically abnormal glands and lack of maturation at the surface. When HGD is diagnosed, aggressive therapies such as mucosectomy, esophageal mucosal resection, radiofrequency ablation (RFA) or even esophageal resection are needed [**29**].

Endoscopic ablative methods are limited due to the risk of metaplasia and even dysplasia that can develop underneath healed squamous epithelium. Neither antireflux surgery nor proton-pomp inhibitor (PPI) can prevent the progression towards cancer in patients with BE, but the regression of dysplasia may be seen, especially after highly selective vagotomy, fundoplication and total duodenal diversion [**30**].

A very low risk of progression was observed in BE patients who initially have no dysplasia and subsequently stay that way. If several endoscopies performed over a period of years confirm the persistence of NDBE there is a small chance of progression to AC [**31**].

Because LGD carries an intermediate risk for the onset of adenocarcinoma, its diagnosis and the related clinical consequences remain more controversial.

Along with the disease duration of more than 10 years, the length of Barrett mucosa, and persistent esophagitis, LGD was identified by a Dutch trial as a risk factor for progression. The absence of any risk factor in nondysplastic Barrett esophagus implied a risk of <1% for progression, whereas those with LGD and one other risk factor had a risk of progression of 18% to 40% [**32**,**33**].

**Molecular markers**

Molecular markers such as p53, cyclin D1 expression and abnormal cellular DNA content by flow cytometry are promising markers associated with carcinogenesis, but none has been proven for routine clinical use [**34**].

Dysplastic BE tissues revealed significantly higher cell proliferation and p53 expression levels compared to BE, reflux esophagitis or BE with concomitant esophagitis. Alterations of cell proliferation and p53 expression showed a strong correlation. Simultaneous activation of cell proliferation and p53 expression strongly suggest their association with esophageal epithelial tumor genesis. Quantification of these parameters in BE is thought to be useful to identify patients at higher risk for progression to adenocarcinoma [**35**].

During the surveillance of Barrett’s esophagus, cytometry from brush cytology, as an add-on to histology, appears to be an additional benefit. Whereas an aneuploid/ intermediate digital image cytometry (DICM) warrants an early re-endoscopy, a diploid DICM underscores the low-risk status especially of patients with low-grade dysplasia [**36**].

Evidence about the effects of surveillance, surveillance intervals, and mortality risk reduction in both NDBE and LGD in BE is still inadequate, but most of the surveillance effort should be concentrated during the first year after a BE patient’s enrolment and on LGD patients [**29**].

**Advanced Imagistic markers**

In an attempt to develop novel endoscopic techniques in order to enhance the detection of dysplasia, there has been a great deal of research over the last years.

The potential advantage might be to enable biopsies to be targeted towards areas containing histological dysplasia and eliminate the need of multiple random sampling.

The benefit would be twofold: 1) better cost-effectiveness due to shorter endoscopies and reduced workload for the pathologist; and 2) improved patient tolerance.

Dye chromoendoscopy, light filtering, and electronic image reprocessing are the three main fields that have been explored so far.

**Chromoendoscopy** (CE) is defined as the topical application of contrast agents to mucosal surfaces within the gastrointestinal (GI) tract in order to enhance the visualization of mucosal details. A variety of agents have been clinically utilized, that can be categorized as absorptive (methylene blue [MB], toluidine blue, Lugol iodine), reactive (Congo red, phenol red), or contrast (indigo carmine [IC]). Mucosal inspection is usually performed by using WLE, but additional modalities such as magnification endoscopy, optical image enhancement (e.g., narrow band imaging [NBI]), and confocal endomicroscopy can be used in order to evaluate suspicious abnormalities.

Even if the use of stains is distinctly low-tech because of the fact that most of the dyes are inexpensive and widely available, CE has been described as an advanced imaging technique [**37**].

The first agent to be effective for the detection of HGD or intramucosal carcinoma in BE was MB. Opposite to MB, IC is not absorbed by the mucosa because it is not a vital stain. IC is simple, easy to use, and has demonstrated its utility in BE surveillance when combined with the magnification endoscopy [**38**].

Acetic acid (AA) is also a widely available, inexpensive, easy to use weak acid that facilitates mucosal contrast enhancement when applied to the surface epithelium so it has been used both with conventional WLE and the magnification endoscopy in order to detect BE and associated dysplasia.

Conflicting results on the utility of MB in dysplasia detection arose. A recent meta-analysis performed by Ngamruengphong et al. concluded that MB does not provide a clinical advantage when compared to the Seattle protocol (random quadrantic biopsies every 2 cm) [**39**].

In a comparative study, high-resolution endoscopy had an equal yield of dysplasia compared to the advanced imaging techniques [**40**].

Low concentration acetic acid (2% to 3%), in contact with the surface epithelium, causes protein denaturation and induces a typical whitening effect on BE mucosa. Increased vascularisation of areas of early neoplasia determines an enhanced and rapid loss of aceto-whitening, which appears as area of redness superposed on a white background. Even if two early randomized studies failed to show the increased detection rate of dysplasia by chromoendoscopy [**41**,**42**], a more recent larger single-center retrospective study has found a higher histological yield in patients who received this agent enhanced chromoendoscopy [**43**]. In order to ascertain whether this agent is a useful adjunct for dysplasia detection, more studies are needed.

**High-definition endoscopy** increases the ability to inspect and visualize subtle mucosal details. Despite significant improvements in image quality, the fundamentals of good endoscopy, particularly careful and thorough inspection by an educated eye, remain the most important tools for dysplasia detection.

**NBI** consists of optical filters controlled by a button switch, which allows one to isolate narrow wavelengths corresponding to the green and blue spectra of light. Light has reduced penetration into the tissues in the blue-green range and therefore helps the visualization of superficial vessels and mucosal pits [**44**].

Even if NBI can prove to be less time consuming and easier to perform in comparison to white light endoscopy, it still remains subject to interobserver variability. In a prospective study with a tandem design, Wolfsen and collaborators [**45**] found that NBI was superior to standard-resolution white light endoscopy with random biopsies in detecting higher grades of dysplasia.

A more recent multicenter randomized crossover study, which compared NBI with high-resolution white light endoscopy, only found a higher histological yield on the per-location analysis but not in the per-patient analysis, suggesting that the clinical overall value of NBI may be limited [**46**].

When compared with the standard approach, NBI required fewer biopsies per patient which may lead to cost savings.

**Autofluorescence imaging** (AFI) uses high frequency blue light, which has the property to excite endogenous fluorophores to emit green fluorescence. Architectural and molecular changes in the columnar mucosa, in the presence of BE with early neoplasia, lead to the reduction of green fluorescence. Therefore, dysplastic lesions can be flagged-up as purple-red areas on a green background. 

An early enthusiasm for the utility of AFI in dysplasia detection was present [**47**,**48**].

Other studies say that this method has a very limited diagnostic value for BE endoscopic surveillance [**49**,**50**].

A multicenter study compared results of AFI targeted biopsies with those of the Seattle protocol. This study found out that AFI positivity correlated with molecular abnormalities of the Barrett’s tissue and even if that area was not dysplastic on a focal biopsy, there was a very high correlation between the molecular read-out from these areas and the overall dysplasia status of the patient. AFI might become a useful tool to direct biopsies for the detection of biomarkers and therefore more objectively determine the risk status of the patient [**51**].

A new technique named **Probe-based confocal laser endomicroscopy** (pCLE) allows the in vivo detection of neoplastic tissue by using a standard endoscope. Incident dysplasia can be more frequently detected by pCLE than by high-definition white light endoscopy HD-WLE in BE. The efficacy of BE surveillance programs could be improve by the higher dysplasia detection rate provided by pCLE [**52**].

A 192 patient international multicenter, prospective, randomized, controlled trial, compared HD-WLE with the Seattle protocol versus HD-WLE plus endoscope-integrated CLE (eCLE) and targeted biopsies [**53**] and found out that the addition of eCLE increased the diagnostic yield for neoplasia from 6% to 22%, with a 4.8-fold reduction in the number of total biopsies required. Still, the main issue of CLE remains the narrow field of view.

The OCT that stands for optical coherence tomography relies on the backscattering of light to obtain cross-sectional images of the tissue and so it enhances the endoscopic image of the superficial layers of the esophagus. The image formation in OCT depends on variations in the reflectance of light from different tissue layers. OCT imaging has demonstrated anatomic structures such as crypts and glands that could potentially permit the endoscopists to diagnose mucosal abnormalities such as BE, including dysplastic changes [**54**,**55**].

## Conclusion

Currently there is not enough evidence in order to recommend advanced imaging modalities for routine Barrett’s surveillance.

The minimum standard should be represented by high-resolution endoscopy and the addition of more complex imaging modalities should be reserved to specialized centers with a high volume of dysplastic cases.

The combination of advanced imaging and molecular biomarkers could represent an improved strategy for a greater stratification of BE patients in the future.
